# Effect of Voacamine upon inhibition of hypoxia induced fatty acid synthesis in a rat model of methyln-nitrosourea induced mammary gland carcinoma

**DOI:** 10.1186/s12860-021-00371-9

**Published:** 2021-06-05

**Authors:** Lakhveer Singh, Manjari Singh, Shubham Rastogi, Anurag Choudhary, Dinesh Kumar, Ritu Raj, Mohd Nazam Ansari, Abdulaziz S. Saeedan, Gaurav Kaithwas

**Affiliations:** 1 Department of Pharmaceutical Sciences, Babasaheb Bhimrao Ambedkar University, VidyaVihar, Raebareily Road, Lucknow, 226025 India; 2 Department of Pharmaceutical Sciences, Assam University, Silchar, Assam 788011 India; 3 Center for Biomedical Research, Sanjay Gandhi Post Graduate Institute of Medical Science, Lucknow, India; 4 Department of Pharmacology, College of Pharmacy, Prince Sattam Bin Abdulaziz University, Al-Kharj, Saudi Arabia

**Keywords:** Prolyl hydroxylase-2, Hypoxia-inducible factor-1α, Fatty acid synthase, Mammary gland carcinoma, Tamoxifen

## Abstract

**Background:**

In the present study, fatty acid synthesis is targeted to combat mammary gland carcinoma by activating prolyl hydroxylase-2 with Voacamine alone and in combination with Tamoxifen. It was hypothesized that the activation of prolyl hydroxylase-2 would inhibit the hypoxia-induced fatty acid synthesis and mammary gland carcinoma. Mammary gland carcinoma was induced with a single dose administration of N-methyl-N-nitrosourea (50 mg/kg,i.p.) and treatment with Voacamine and Tamoxifen 15 days after carcinogen administration.

**Results:**

At the end of the study, hemodynamic profiling of animals was recorded to assess the cardiotoxic potential of the drug. Blood serum was separated and subjected to nuclear magnetic resonance spectroscopy. Carmine staining and histopathology of mammary gland tissue were performed to evaluate the anti-angiogenic potential of the drug. The antioxidant potential of the drug was measured with antioxidant markers. Western blotting was performed to study the effect of the drug at the molecular level.

**Conclusion:**

Results of the study have shown that Voacamine treatment stopped further decrease in body weight of experimental animals. The hemodynamic study evidenced that Voacamine at a low dose is safe in cardiac patients. Microscopic evaluation of mammary gland tissue documented the anti-angiogenic potential of Voacamine and Tamoxifen therapy. Perturbed serum metabolites were also restored to normal along with antioxidant markers. Immunoblotting of mammary gland tissue also depicted restoration of proteins of the hypoxic and fatty acid pathway. Conclusively, Voacamine and its combination with Tamoxifen activated prolyl hydroxylase-2 to combat mammary gland carcinoma.

## Background

Mammary gland carcinoma originates in breast tissue cells, particularly in the lobules (milk glands) or in the ducts, which connect the lobules with the nipple [1]. Uncontrolled dividing of these cells forms a mass or lump at the site of their origin, transforming itself into malignant neoplasm if it remains undiagnosed [2–4]. In most cases, it remains asymptomatic when the tumor size is small. Moreover, when the tumor size increases and becomes symptomatic, it becomes irresponsive to radiotherapy and chemotherapy due to the development of hypoxia, a condition characterized by low oxygen [5]. Tamoxifen (TMX) is an approved drug for mammary gland carcinoma, and the presence of estrogen receptors (ER) on the cancer cells is a must for its activity. But under hypoxia, cancer cells either do not express or express a significantly lower number of ERs on their surface [6]. Hence, TMX fails to illicit its cytotoxic effect in solid hypoxic malignant tumors of the mammary gland.

Tumor hypoxia increases resistance to chemotherapy and alters the metabolism of glucose and fatty acids [7]. Various studies have reported that hypoxia enhances the de novo fatty acid synthesis to accomplish plasma membrane synthesis of rapidly dividing cancer cells [8–10]. Some studies also said that de novo fatty acid synthesis fails to meet the increasing demand for fatty acids [11]. Therefore cancer cells undergo some changes to enhance the uptake of dietary lipids. It is also reported that uptake of dietary fatty acid also increases under hypoxia [12]. Therefore, de novo synthesized fatty acids and fatty acids derived from nutritional sources help furbish tumor cells under hypoxia [13].

Moreover, fatty acids also have a role in energy production and signal transduction [14]. Fatty acids also play an essential role in the angiogenesis of hypoxic cancerous cells. A recent study conducted by Bruning et al. has established a relationship between fatty acid synthase (FASN-prominent enzyme in fatty acid synthesis pathway) overexpression and angiogenesis. Inhibition of FASN reduced angiogenesis in experimental animals as evidenced by reduced branching of vascular tissue [15]. Now, it is crystal clear that hypoxia-activated hypoxia-inducible factor-1α (HIF-1α) reprograms the fatty acid metabolism that can be inhibited by downregulating the HIF1-α.

After thoroughly studying the hypoxic pathway, it was observed that prolyl hydroxylase-2 (PHD-2) is an oxygen-dependent negative regulator of HIF-1α. It becomes irresponsive in the absence of oxygen in solid tumors mammary gland, and thus HIF-1α escapes from proteolytic degradation [16, 17]. Chemical activation of PHD-2 in solid tumors of the mammary gland could curtail all the effects of HIF-1α (glycolysis, fatty acid synthesis, protein synthesis, and angiogenesis).

In the present study, author upregulated PHD-2 activity with Voacamine (VOA) to inhibit hypoxia-induced fatty acid synthesis in mammary gland carcinoma of albino Wistar rats. The study also delineates the role of VOA and TMX combination therapy on mammary gland carcinoma cells. It was hypothesized that VOA would enhance TMX action by decreasing HIF-1α and increasing ER on breast carcinoma cells.

## Results

### Virtual screening of compounds

**Fig. 1:**
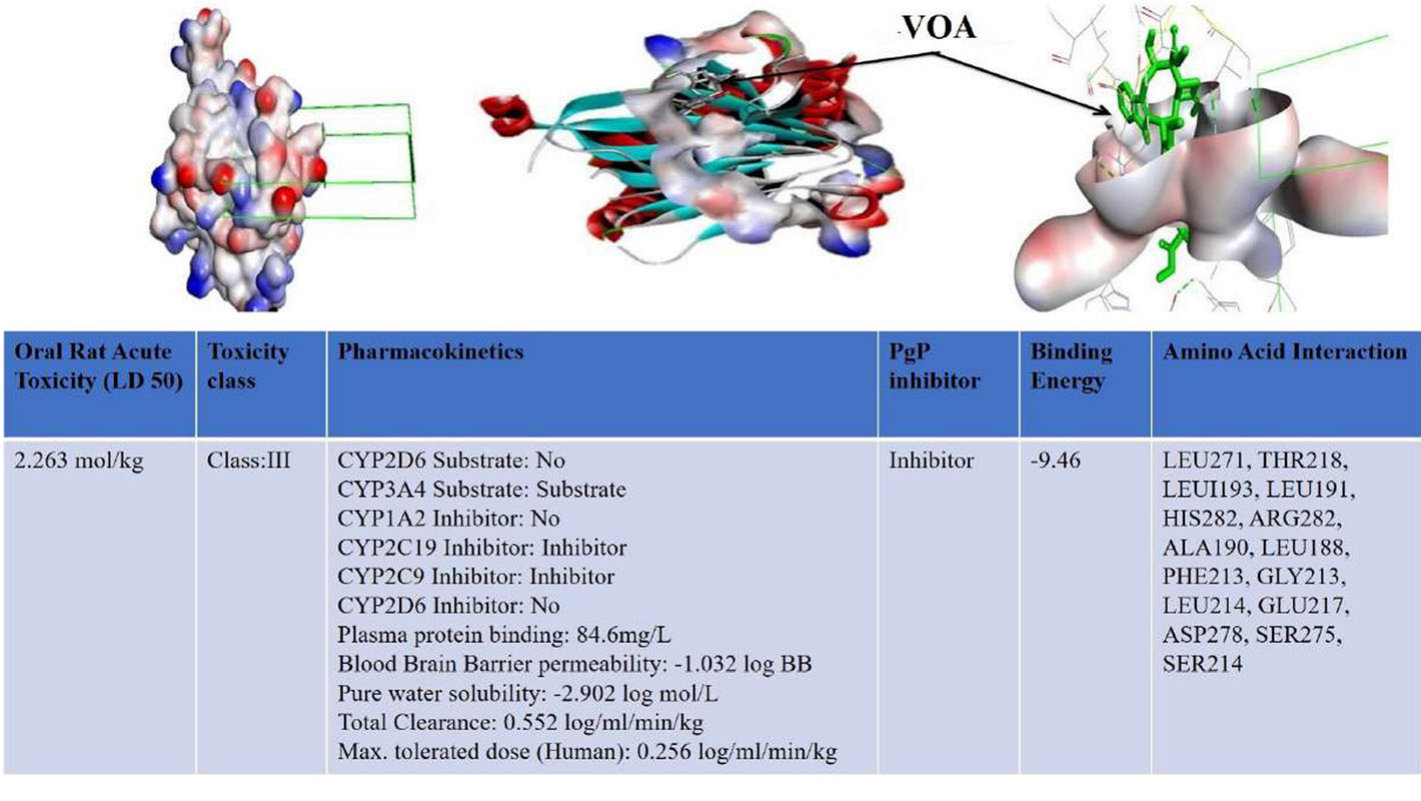
Docked pose of VOA with PHD-2 and in silico pharmacokinetics. VOA was docked with PHD2 protein (PDB ID: 2G19) with Autodock 4.2, and calculated binding energy was found to be −9.46 kCal/mol. The drug has hydrogen bonding interaction with LEU271, THR218, LEU193, LEU191, HIS282, ARG282, ARG281, ALA190, LEU188, PHE213, GLY213, LEU214, GLU217, ASP278, SER275, and SER214 type of amino acids. In silico*,* ADME was tested with pkCSM software (http://biosig.unimelb.edu.au/pkcsm/prediction_single/adme_1604411699.32), and results are presented in Table 1

In docking studies, calculated negative energy determines the strength of binding and affinity between the docked ligand and the protein [18]. When VOA docked with PHD-2, the maximum binding energy was found to be − 9.46 kcal/mol **(**Fig. 1**)**. The docked pose of the VOA had interaction with a huge number of amino acids (LEU271, THR218, LEU193, LEU191, HIS282, ARG282, ARG281, ALA190, LEU188, PHE213, GLY213, LEU214, GLU217, ASP278, SER275, and SER214) which further support the stability of the protein-ligand complex. After docking studies, VOA was subjected to the calculation of rodent oral toxicity, and the calculated LD50 was found to be 2.263 mol/kg. The compound belongs to the class-III toxicity class. VOA was further subjected to in silico Absorption, distribution, metabolism and excretion (ADME) studies following toxicity studies **(**Fig. 1**,** Table 1**)**. The pharmacokinetic profile of the drugs shows that the drug has high plasma protein binding (84.588839) and has very less solubility in pure water (water solubility− 2.902 log mol/L). The volume of distribution in humans is 0.808 log L/kg, as calculated by the software. The blood brain barrier (BBB) permeability of the drug was found to be − 1.032 log BB. VOA inhibited CYP2C19 and CYP2C9 liver enzymes. The drug is also a substrate but not an inhibitor of CYP3A4. The drug has shown significant hepatotoxic potential. The drug VOA is neither a substrate and nor an inhibitor of CYP2D6. The total renal clearance was found to be 0.552 log ml/min/kg, and the maximum tolerated dose in humans was found to be 0.256 log mg/kg/day. The drug also inhibited an ether-a-go-go-related gene (hERG1&II), indicating a significant cardiotoxic effect. VOA has the potential to inhibit PgP-the efflux pump. Thus, it can potentiate the action and increase the toxicity of all those effluxes through this pump, like doxorubicin, Vincristine, and TMX [19].

**Table 1:** In Silico ADME studies

Property	Model Name	Predicted Value	Unit
Absorption	Water solubility	**-2.902**	Numeric (log mol/L)
Absorption	Caco2 permeability	**1.292**	Numeric (log Papp in 10^−6^ cm/s)
Absorption	Intestinal absorption (human)	**100**	Numeric (% Absorbed)
Absorption	Skin Permeability	**−2.735**	Numeric (log Kp)
Absorption	P-glycoprotein substrate	**Yes**	Categorical (Yes/No)
Absorption	P-glycoprotein I inhibitor	**Yes**	Categorical (Yes/No)
Absorption	P-glycoprotein II inhibitor	**Yes**	Categorical (Yes/No)
Distribution	VDss (human)	**0.808**	Numeric (log L/kg)
Distribution	Fraction unbound (human)	**0.432**	Numeric (Fu)
Distribution	BBB permeability	**−1.032**	Numeric (log BB)
Distribution	CNS permeability	**−1.851**	Numeric (log PS)
Metabolism	CYP2D6 substrate	**No**	Categorical (Yes/No)
Metabolism	CYP3A4 substrate	**Yes**	Categorical (Yes/No)
Metabolism	CYP1A2 inhibitor	**No**	Categorical (Yes/No)
Metabolism	CYP2C19 inhibitor	**Yes**	Categorical (Yes/No)
Metabolism	CYP2C9 inhibitor	**Yes**	Categorical (Yes/No)
Metabolism	CYP2D6 inhibitor	**No**	Categorical (Yes/No)
Metabolism	CYP3A4 inhibitor	**No**	Categorical (Yes/No)
Excretion	Total Clearance	**0.552**	Numeric (log ml/min/kg)
Excretion	Renal OCT2 substrate	**No**	Categorical (Yes/No)
Toxicity	AMES toxicity	**Yes**	Categorical (Yes/No)
Toxicity	Max. tolerated dose (human)	**0.256**	Numeric (log mg/kg/day)
Toxicity	hERG I inhibitor	**No**	Categorical (Yes/No)
Toxicity	hERG II inhibitor	**Yes**	Categorical (Yes/No)
Toxicity	Oral Rat Acute Toxicity (LD50)	**2.263**	Numeric (mol/kg)
Toxicity	Oral Rat Chronic Toxicity (LOAEL)	**−0.32**	Numeric (log mg/kg_bw/day)
Toxicity	Hepatotoxicity	**Yes**	Categorical (Yes/No)
Toxicity	Skin Sensitisation	**No**	Categorical (Yes/No)
Toxicity	*T.Pyriformis* toxicity	**0.285**	Numeric (log ug/L)
Toxicity	Minnow toxicity	**−0.218**	Numeric (log mM)

### Effect of VOA/TMX therapy on cardiac function of experimental animals

**Fig. 2:**
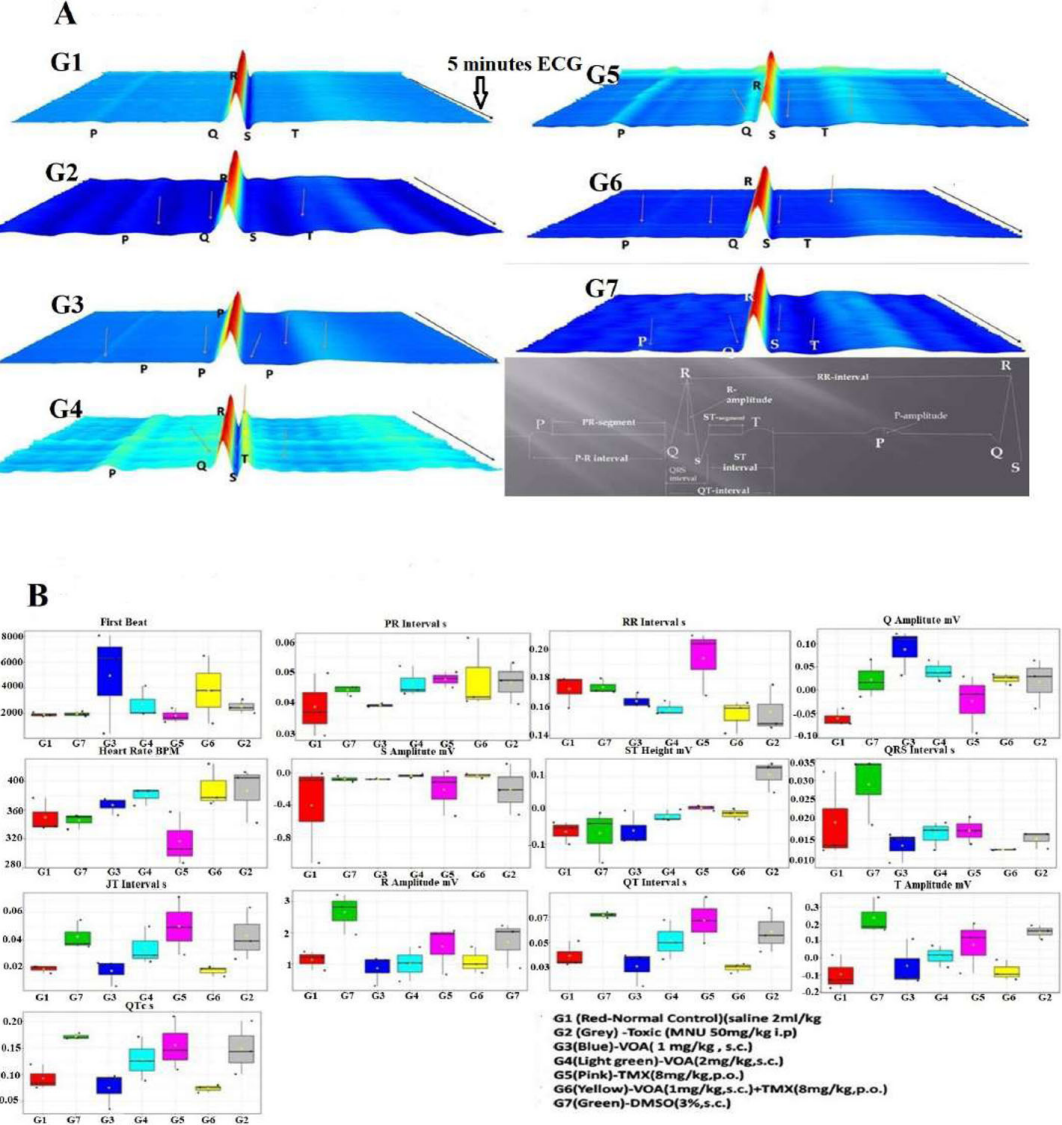
**A**: Waterfall map presentation of 5 min ECG recording of experimental animals. G1(normal control receives normal saline 2 ml/kg, p.o.); G2 (toxic control receives MNU 50 mg/kg, i.p); G3(MNU 50 mg/kg, i.p. + VOA 1 mg/kg, s.c.); G4 (MNU 50 mg/kg, i.p. + VOA 2 mg/kg, s,c.); G5 (MNU 50 mg/kg, i.p. + TMX 8 mg/kg, p.o.); G6 (MNU 50 mg/kg,i.p. + VOA 1 mg/kg, s.c. + TMX 1 mg/kg s.c.) and G7 (dummy control receives 3% DMSO solution s.c.). **B**: Box –Cum whisker plot of ECG recording of VOA treated on experimental animals. Red-G1-Normalcontrol (saline 2 ml/kg), Grey-G2-Toxic control-MNU (50 mg/kg, i.p), Blue-G3-VOA low dose (1 mg/kg, s.c), Light green-G4-VOA high dose (2 mg/kg, s.c.), Pink-G5-TMX (8 mg/kg,p.o.) Yellow-G6- MNU (50 mg/kg, i.p.) + VOA (1 mg/kg,s.c.) + TMX (8 mg/kg,p.o.), GreenG7-DMSO 3% (3 ml/kg, s.c)

Since in silico studies have shown, VOA is an inhibitor of hERG genes that warns against its cardiotoxic potential. Therefore, we performed an electrocardiogram (ECG) of experimental animals to assess cardiotoxicity, if any, with the current treatment drugs [20]. Normal ECG was recorded in normal group animals as the only vehicle was administered until the end of the study. But a significant change in ECG parameters like increase in PR interval, Q amplitude, HR, ST height, JT interval, R amplitude, QT interval, T amplitude, QTc interval observed after MNU administration (Fig. 2A and B). A sharp increase in Q amplitude, slight increase in HR and T amplitude was observed in VOA low dose treated group, whereas no significant change in other parameters was observed. But on treatment with a high dose of VOA, a very small reduction in PR interval, HR, ST height, JT interval, QT interval, T amplitude, and QTc interval was observed compared to the toxic control. Standard therapy with TMX significantly increased the PR interval, RR interval, Q amplitude, ST height, QRS complex, JT interval, QT intervals, T amplitude, and QTc intervals, but a sharp decrease in HR was noted when compared with normal rats. Combination therapy of VOA and TMX caused a sharp decrease in RR interval, Q-amplitude, HR, and ST height while decreased RR interval, JT height, and R amplitude. A significant change in QRS interval, JT interval amplitude, QT interval amplitude, and QTc interval was noted with dimethyl sulphoxide (DMSO) administration compared with normal control. All other groups like toxic control, standard group, and DMSO group were considered a similar trend in ECG parameters were observed.

Heart rate variability (HRV) analysis of toxic control rats showed decreased average RR and average rate compared to other treatment groups. No significant change in SD rate, SDRR, LF, HF, and LF/HF ratio were noted (Table 2).

**Table 2:** Effect of VOA on HRV of rats

Groups	G1	G2	G3	G4	G5	G6	G7
**Time domain**							
**Average RR**	161.65 ± 3.46	146.90 ± 1.83	172.15 ± 11.10***	160.8 ± 0.56	155.6 ± 0.42	291.15 ± 34.011***	160.9 ± 2.40
**SD RR**	3.18 ± 2.73	2.39 ± 1.45	2.22 ± 1.64	7.68 ± 0.13*	5.34 ± 5.36	6.42 ± 0.91	4.18 ± 0.465
**Average Rate**	371.4 ± 7.63	408.6 ± 5.23	22.55 ± 1.27***	386.35 ± 0.21**	386.35 ± 0.21	207.55 ± 24.25***	373.15 ± 5.44***
**SD Rate**	7.061 ± 5.79	6.70 ± 4.15	4.287 ± 2.90	18.35 ± 0.388*	13.367 ± 13.45	4.53 ± 0.36	9.50 ± 0.56
**Frequency domain**							
**VLF**	44.97 ± 20.194	36.23 ± 34.23	60.46 ± 6.68	45.66 ± 9.079	52.79 ± 54.92	49.48 ± 3.40**	62.41 ± 4.27
**LF**	5.85 ± 4.48	4.32 ± 2.46	5.96 ± 0.73	6.31 ± 1.24	4.97 ± 3.98	20.28 ± 14.03	7.93 ± 5.44
**HF**	39.53 ± 21.44	31.53 ± 8.37	26.20 ± 7.38	45.45 ± 7.22	39.82 ± 49.04	24.96 ± 11.92	26.52 ± 1.03
**LH/HF**	0.13 ± 0.038	0.14 ± 0.11	0.24 ± 0.095	0.13 ± 0.004	0.24 ± 0.20	1.06 ± 1.07*	0.29 ± 0.22

Values are presented as Mean ± SD, each group contains 6 animals. Comparisons were made on the basis of the one-way ANOVA followed by Bonferroni test. All groups were compared to the toxic control group (*p < 0.05, **p < 0.01, ***p < 0.001)

### Effect of VOA therapy on angiogenesis

**Fig. 3:**
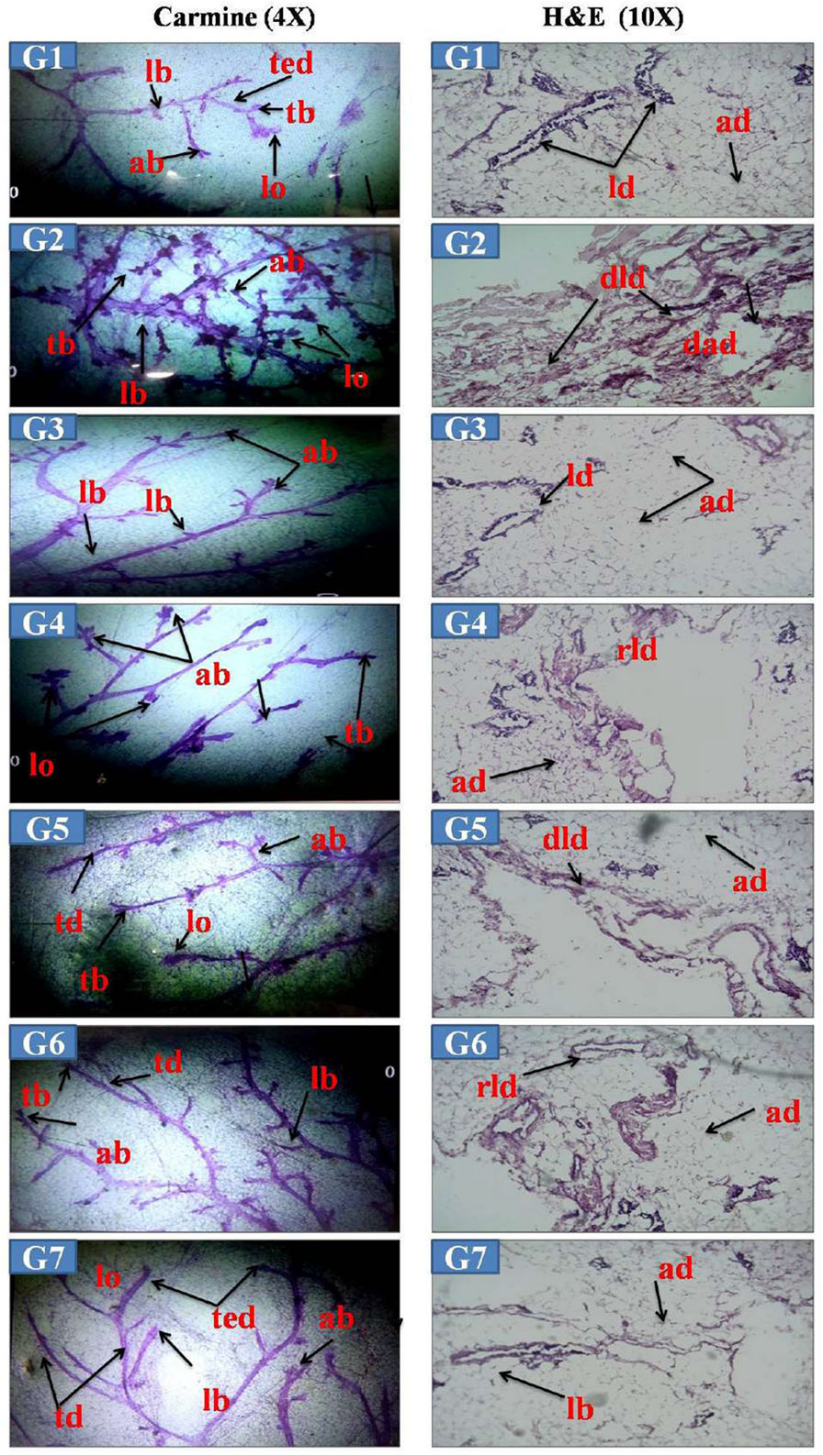
Effect of VOA on the morphology of mammary gland carmine staining. G1&G7-Carmine staining of normal control (G1) and DMSO (G7) administered rats showing very less branching of ducts, **terminal end duct (ted), terminal bud (tb),** normal **lateral bud (lb), alveolar buds** (**ab), B**-Carmine staining toxic control (G2) rats showing excessive branching of mammary gland ducts, a high number of **ab** and **lobules (lo**) along with **tb** and **lb**. **C-G3** rats treated with a low dose of VOA showing very less branching of mammary gland ducts and few numbers of **ab** and no **lo** formation was noted, **D-G4**-Rats treated with a high dose of VOA showing **tb, lb, ab,** and **lo**, **E-G5**-Rats treated with TMX showing **lb, tb, ab,** and lo, **F-G6**-Rats treated with combination therapy of VOA and TMX showing along with **tb, lb, ted** and **ab** only. **H&E staining: G1-G7 -**Histology of G1 and G7 rats were showing the normal shape and of lactiferous duct (ld) and adipocytes, B-G2-Histology of rats showing **damaged lactiferous duct (dld)** and distorted shape of **adipocytes (ad**), C-G3-Rats were showing normal shape of ld, and ad, D-G4-VOA high dose treated rats showing regenerated **lactiferous duct (rld**) and **ad**, E-Rats treated with TMX showing **rld** and ad, F-G6-VOA and TMX treated rats showing the normal shape of **ld** and **ad**

Angiogenesis is the characteristic feature of malignant tumors. Therefore, carmine staining was performed to study the anti-angiogenic effect of the VOA therapy. The change in the development of micro ducts of mammary glands was compared in toxic and control animals. Carmine staining of normal control animals witnessed the normal growth and differentiation of mammary glands tissue. A very few no. of lobules (lo), alveolar buds (ab), terminal end duct (ted), lateral bud (lb), and terminal bud (tb) were observed in this group as the only vehicle was administered up to the end of study (Fig. 3 G1-G7). No significant change in DMSO treated group was observed. Excessive branching of mammary gland ducts, tb, lb., high no. of ab and lb formation was observed in the MNU treated animals compared with normal controls animals. A significant reduction in lb., tb, ab, and lo formation was observed after VOA low dose and high dose of VOA. Also, a better anti-angiogenic potential was observed with a high dose. Standard therapy also worked well and imparted mammary gland protection comparable to the high dose of VOA. Combination therapy VOA with TMX showed anti-angiogenic potential comparable to the low dose of VOA, i.e., very less branching of mammary gland ducts, and few numbers of lo was observed.

### Histopathological analysis of mammary gland tissue after VOA treatment

Further, H&E staining of mammary gland tissues was performed to validate the results of carmine staining. Normal shape and structure of lactiferous duct and adipocytes were noted in the normal group animals (Fig. 3G1-G7). Significant damage to the lactiferous duct (dld) and distorted shape of adipocyte (dad) were manifested in MNU treated animals. Treatment with a low dose of VOA imparted significant protection to the mammary gland tissue as the normal architecture of adipocytes and lactiferous ducts comparable to the normal control animals were observed in this group. Histology of VOA high dose treated animals also imparted protection evidenced by the appearance of some of the normal lactiferous duct but not up to the extent of low dose. Standard therapy with TMX imparted very little protection as evidence by large damage to ld and da. Significantly, good results were observed with combination therapy compared to the standard therapy as evidenced by the regeneration of lactiferous duct (rld) and normal shape of adipocytes.

### Changes recorded upon the bodyweight of animals after VOA treatment

**Fig. 4:**
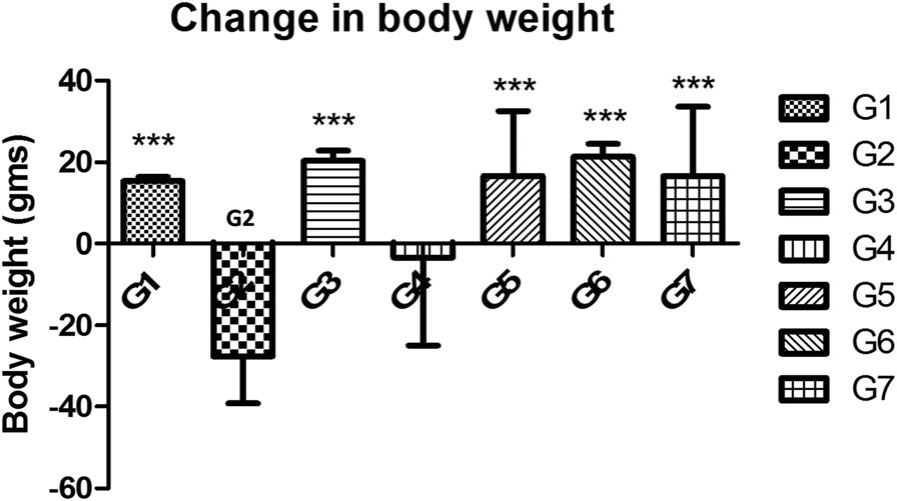
Effect of VOA/TMX on body weight of experimental animals. Cachexia is the hallmark of cancer. Continuous loss in body weight after MNU treatment was observed in toxic control (G2). Treatment with a low dose of VOA (G3), TMX treatment (G5), and combination therapy of VOA and TMX (G6) stopped the further loss of body weight like normal control rats (G1, G7). Continuous loss in body weight was also observed in VOA high dose (2 mg/kg,s.c.) treatment which indicates VOA has toxic consequences. G1(normal control receives normal saline 2 ml/kg, p.o.); G2 (toxic control receives MNU 50 mg/kg, i.p); G3(MNU 50 mg/kg, i.p. + VOA 1 mg/kg, s.c.); G4 (MNU 50 mg/kg, i.p. + VOA 2 mg/kg, s,c.); G5 (MNU 50 mg/kg, i.p. + TMX 8 mg/kg, p.o.); G6 (MNU 50 mg/kg,i.p. + VOA 1 mg/kg, s.c. + TMX 1 mg/kg s.c.) and G7 (dummy control receives 3% DMSO solution s.c)

Cachexia is very common in cancer patients due to the alteration of metabolism. Therefore, in the next step, we determined the bodyweight of experimental animals up to the end of the study. The percentage decrease in body weight was calculated and shown in Fig. 4. A continuous increase in body weight of normal control and DMSO treated animals was observed as the only vehicle was administered up to the end of the study. A significant decrease in body weight of toxic control animals was recorded in comparison to the control animals. Upon treatment with a low dose of VOA, a substantial increase in animals’ bodyweight was noted. Still, treatment with a high dose of VOA caused an unexpected decrease in the bodyweight of animals like toxic control. TMX being a well-known anticancer drug, stopped further decrease in body weight of animals, instead of increased compared to normal controls. Combination therapy with VOA and TMX also worked well and provided significant protection from decreasing body weight.

### Effect of VOA on antioxidant markers

Next, we assessed the free radical scavenging mechanism of MNU treated rats because failure to neutralize free radicals can damage DNA and mammary gland carcinoma. Excessive free radical antioxidant markers like thiobarbituric acid reactive substances (TBARs), protein carbonyl (PC), superoxide dismutase (SOD), catalase, and glutathione (GSH) plays an essential role by neutralizing free radical generated in the body (Table 3). The level of these can increase or decrease in deseased conditions. A significant increase in TBARs and PC level was observed in toxic control (868.6 ± 67.31, 57.08 ± 7.34) when compared with the normal control (479.70 ± 56.4***, 21.30 ± 6.4***). When therapy was started with a low dose of VOA, a significant moderate decrease in TBARs and PC was observed with VOA high dose and standard therapy.

**Table 3:** Effect of VOA and TMX on antioxidant markers

Groups	TBAR’s (nm of MDA/μg of protein	GSH (mg %)	SOD (units of SOD/mg of protein)	Catalase (nm of H_**2**_O_**2**_/ min/mg of protein)	PC nmol/mg of protein
G1 [Normal control, normal saline 2 ml/kg, p.o.]	489.70 ± 56.4***	0.71 ± 0.3***	0.03 ± 0.01***	0.43 ± 0.2***	21.30 ± 6.4***
G2 [toxic control, MNU 50 mg/kg, i.p]	868.6 ± 67.31	0.63 ± 0.03	0.022 ± 0.00	0.19 ± 0.036	57.08 ± 7.34
G3 [MNU 50 mg/kg, i.p. + VOA 1 mg/kg, s.c.]	304.5 ± 54.9***	1.4 ± 0.09***	0.037 ± 0.00**	0.34 ± 0.02*	24.4 ± 3.4***
G4 [MNU 50 mg/kg, i.p. + VOA 2 mg/kg, s,c.]	698.56 ± 90***	0.72 ± 0.093	0.021 ± 0.00	0.28 ± 0.06	45.3 ± 0.4
G5 [MNU 50 mg/kg, i.p. + TMX 8 mg/kg, p.o.]	592.4 ± 25.8***	0.80 ± 0.09	0.034 ± 0.001**	0.34 ± 0.12	35.6 ± 0.6
G6 [MNU 50 mg/kg,i.p. + VOA 1 mg/kg, s.c. + TMX 1 mg/kg s.c.]	207.5 ± 54.9***	1.9 ± 0.09***	0.07 ± 0.00**	0.56 ± 0.06*	27.3 ± 0.4***
G7 [dummy control, 3% DMSO solution s.c]	379.70 ± 56.4***	0.71 ± 0.3***	0.06 ± 0.01*	0.45 ± 0.2***	23.30 ± 6.4***

Values are presented as Mean ± SD, each group contains 6 animals. Comparisons were made on the basis of the one-way ANOVA followed by Bonferroni test. All groups were compared to the toxic control group (*p < 0.05, **p < 0.01, ***p < 0.001)

SOD, catalase, and GSH levels were significantly reduced in the toxic control (0.022 ± 0.001, 0.19 ± 0.03, 0.63 ± 0.03) group. Treatment with a low dose of VOA significantly increased SOD, catalase, and GSH (0.037 ± 0.004**, 0.34 ± 0.02*). Only a moderate increase in SOD, catalase, and GSH was observed with a high dose of VOA. Standard therapy with TMX significantly raised the level of SOD, catalase, and GSH (0.034 ± 0.005**, 0.34 ± 0.12, 0.80 ± 0.09). The combination of VOA and TMX worked well and praised the level of SOD, catalase, and GSH (0.07 ± 0.004**, 0.56 ± 0.06*, 1.9 ± 0.09***) considerably.

### Metabolomics changes after treatment with VOA

**Fig. 5:**
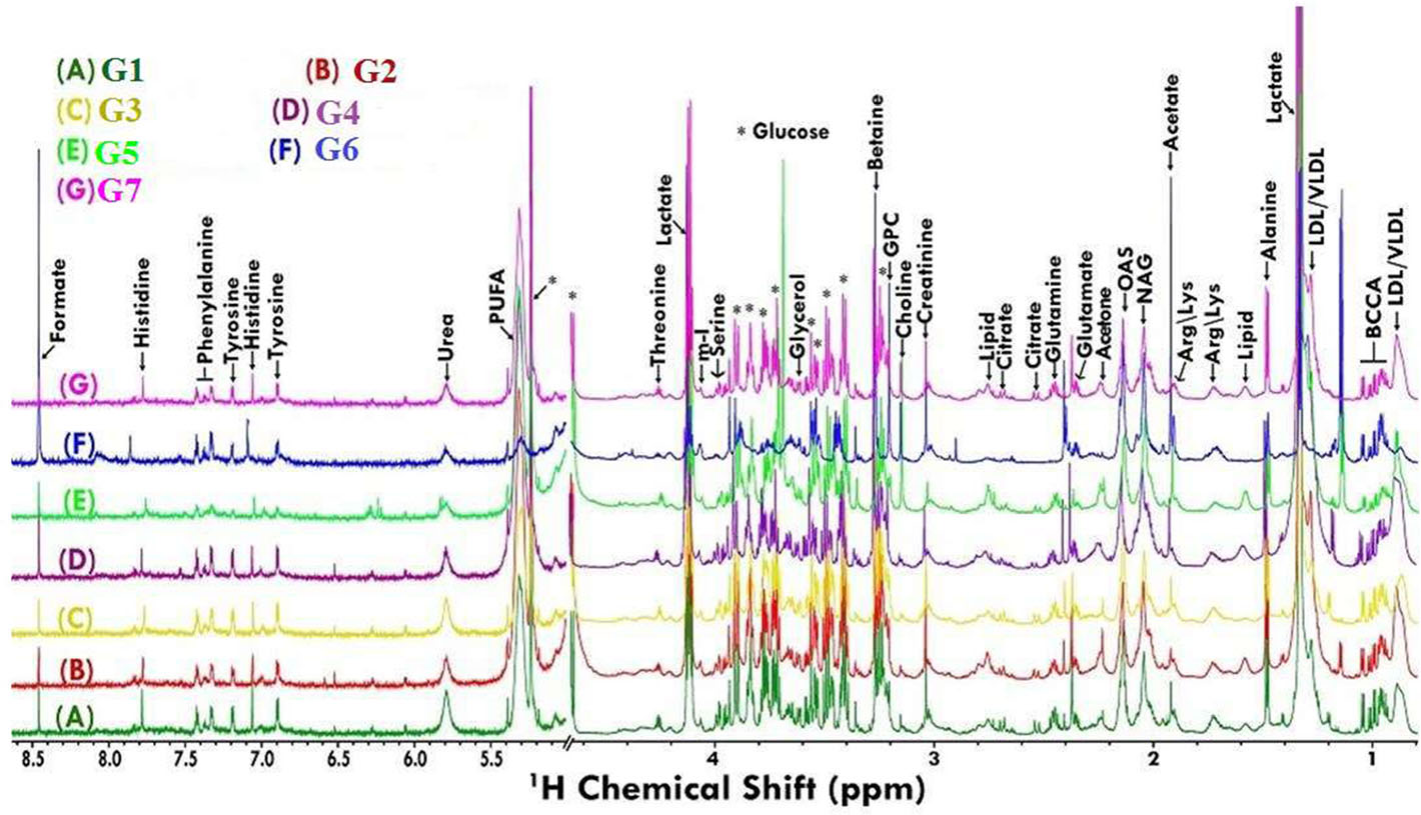
Stack plot representation of H ^1^ NMR peaks of serum metabolites. The peaks annotated in the figure show the assignments of serum metabolites. The abbreviations used are: LDL/VLDL: Low/very-low-density lipoproteins; PUFA: polyunsaturated fatty acids; Ile: isoleucine; Leu: leucine; Val: Valine, Pyr: pyruvate; Ch: choline; GPC: glycerophosphocholine; Glucose resonances have been indicated using symbol asterisk “*”

**Fig. 6:**
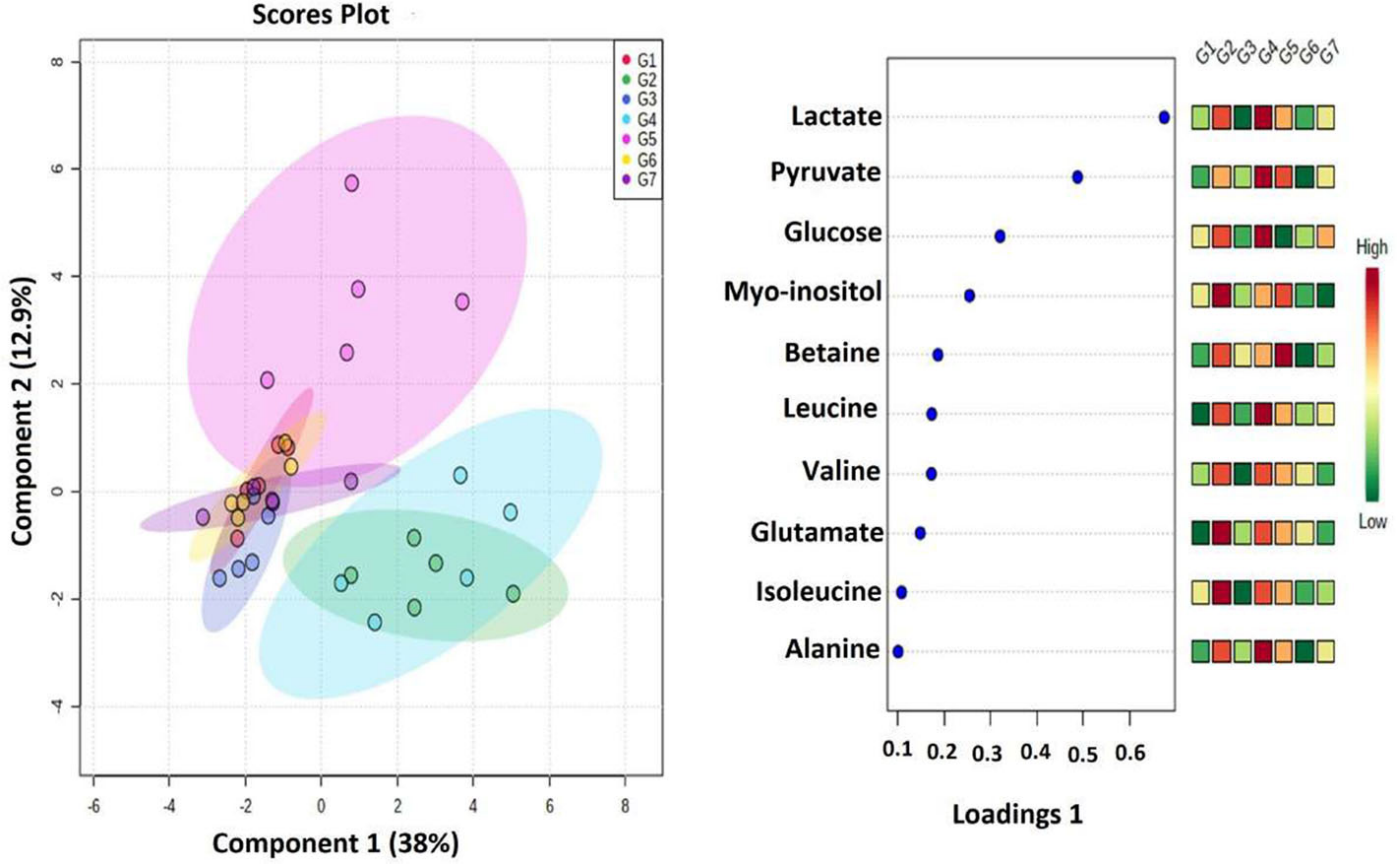
2D-PLS-DA score plot of rat serum metabolites treated with VOA and TMX. The recorded serum metabolomics data were first analyzed with combined 2D PLS-DA to see the variation in the principal components. The 2D PLS score plots of toxic control (Group 2) and that Group 4 animals treated with MNU dose depicting a clear separation from the normal, VOA low dose (G3), TMX treated (Group 5), and with a combination of both of these drugs (Groups 6). A metabolic profile of Group 4 animals was observed like that of toxic control animals after treatment with a high dose of VOA, which indicates its toxicity at this dose. This means VOA low dose and its combination with somehow resetting back the perturbed metabolites due to MNU administration excluded from the data matrix to evaluate the discriminatory significance of aromatic residues

**Fig. 7:**
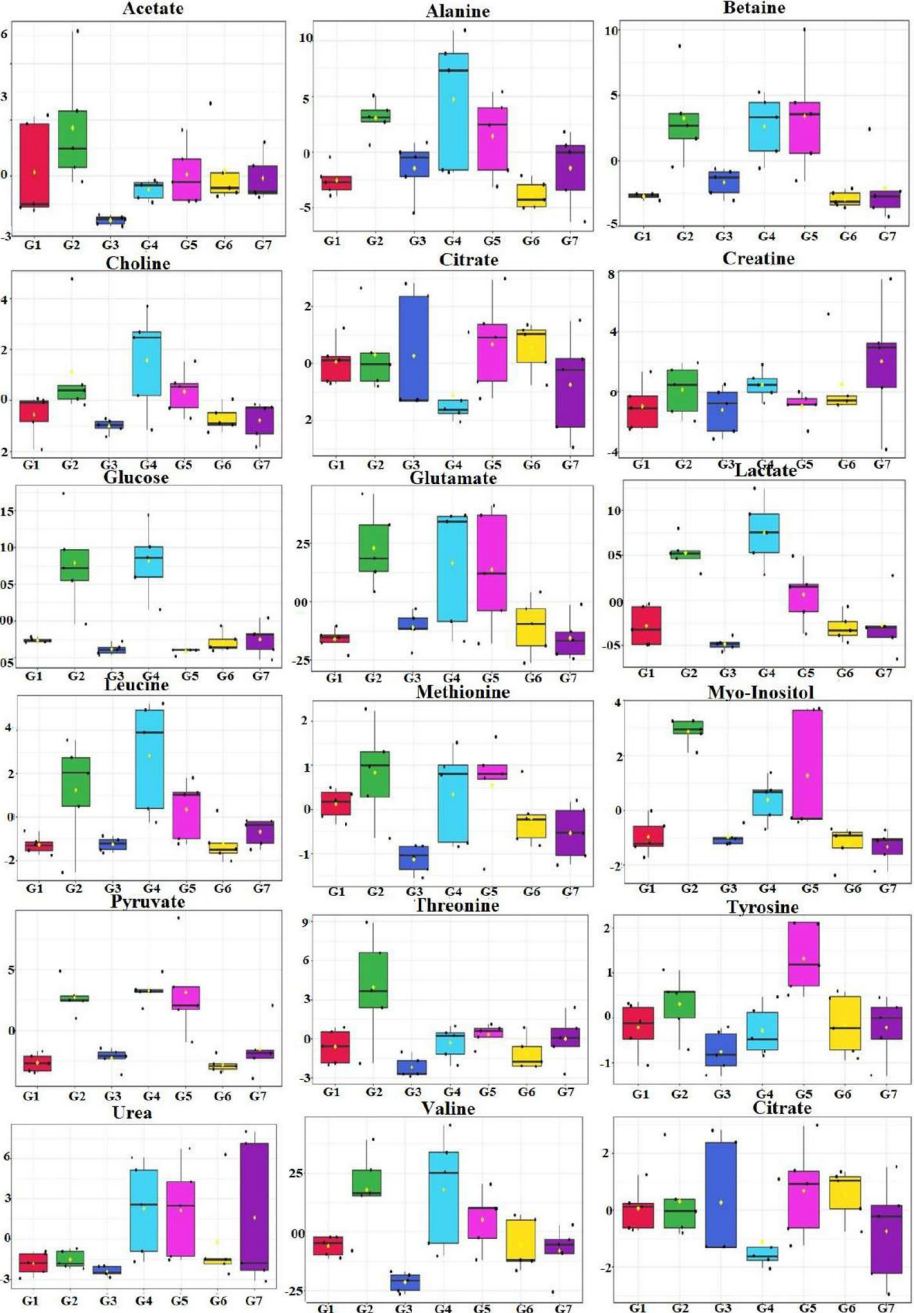
Box cum whisker plot of rat serum metabolites of experimental animals after treatment with VOA/TMX. Representative box-cum whisker plots showing the quantitative variation of relative signal integrals for serum metabolites. For presented metabolite entities, the VIP score > 1 and statistical significance are at the level of *p* ≤ 0.05. In the box plots, the boxes denote the interquartile ranges; horizontal lines inside the boxes denote the median and bottom and top boundaries of boxes are 25th and 75th percentiles, respectively. Lower and upper whiskers are 5th and 95th percentiles, respectively

Having determined the oxidative stress in experimental animals, next, we analyzed their serum metabolomics profile because a stressed tissue produces abnormal serum metabolites. Purposely, serum samples from all the groups were collected and subjected to H^1^ nuclear magnetic resonance (NMR) analysis to compare the metabolomics profile of experimental animals (Figs. 5 and 6). Thirty-four metabolites were analyzed in the serum samples. Major perturbation was observed in glucose, lactate, pyruvate and citrate along with amino acids leucine, isoleucine threonine, tyrosine, and proline. A significant change in glucose, pyruvate, lactate, citrate, succinate, leucine, isoleucine, threonine, tyrosine, and proline in MNU treated toxic control rats were observed when compared to the normal rats (Fig. 7). A significant reduction in glucose, pyruvate, lactate, citrate succinate, amino acids threonine, leucine, isoleucine, and tyrosine was observed with a low dose VOA. Still, the opposite trend in these metabolites was observed with a high dose of VOA. A minimal decrease in metabolites mentioned above was observed with the standard therapy. The combined therapy of VOA with TMX worked very well, reduced serum concentration of glucose, lactate, pyruvate, citrate, succinate, threonine tyrosine, leucine, isoleucine like VOA low dose treatment. A normal metabolic profile was observed with DMSO treatment. No significant alteration in the above metabolite was observed when compared with normal control upon treatment with DMSO.

### Western blotting

**Fig. 8:**
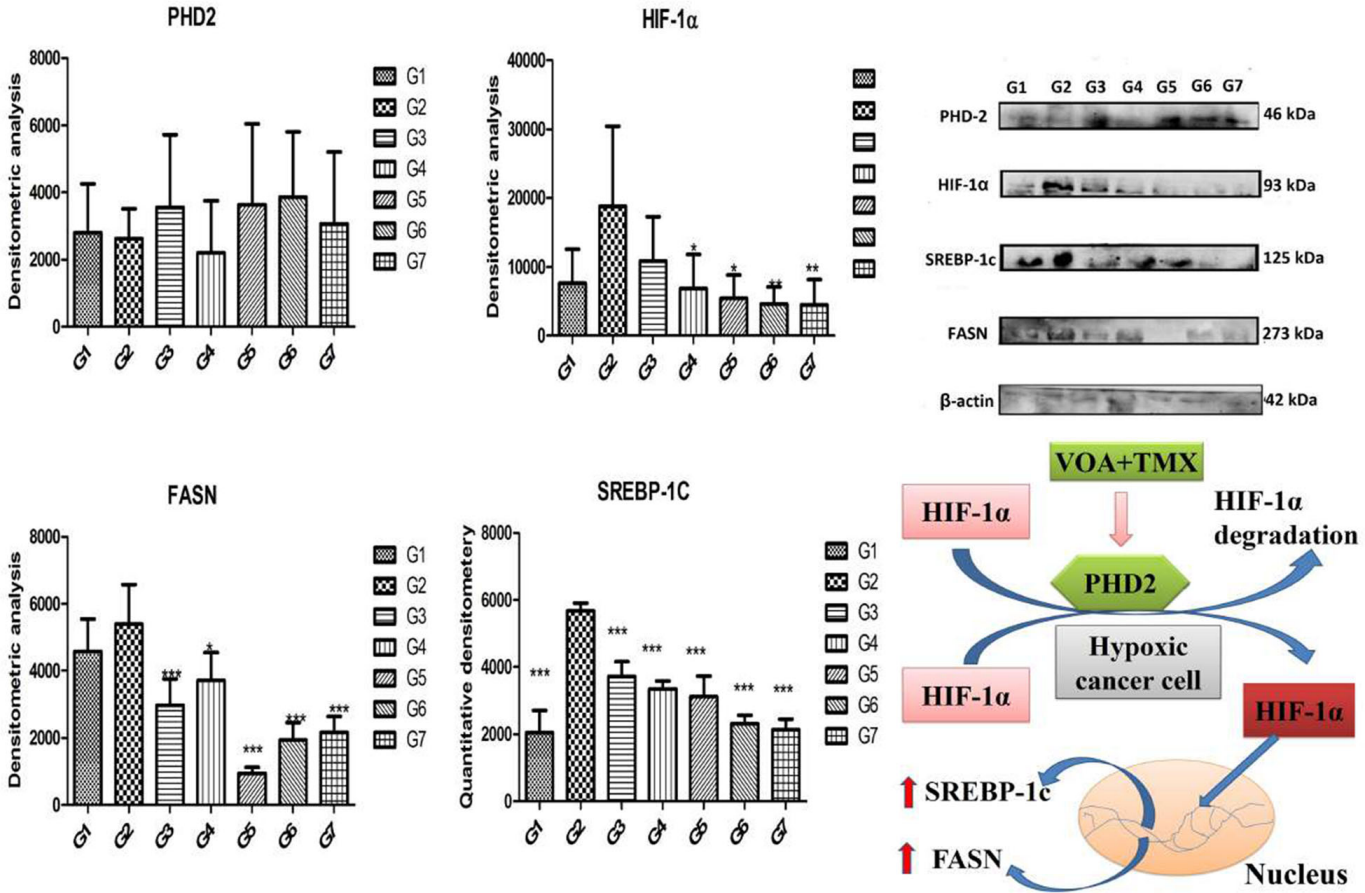
Effect of VOA/TMX on proteins of the hypoxic pathway. Hypoxia in cancer cells activated HIF-1α, which enhanced the cellular expression of SREBP-1c and FASN and thus fatty acid synthesis in toxic control. PHD-2 is a negative regulator of HIF-1α, and its expression is downregulated in a hypoxic environment. Treatment with VOA upregulated PHD-2 activity and downregulated HIF-1α and reverted its downstream effects. G1(normal control receives normal saline 2 ml/kg, p.o.); G2 (toxic control receives MNU 50 mg/kg, i.p); G3(MNU 50 mg/kg, i.p. + VOA 1 mg/kg, s.c.); G4 (MNU 50 mg/kg, i.p. + VOA 2 mg/kg, s,c.); G5 (MNU 50 mg/kg, i.p. + TMX 8 mg/kg, p.o.); G6 (MNU 50 mg/kg,i.p. + VOA 1 mg/kg, s.c. + TMX 1 mg/kg s.c.) and G7 (dummy control receives 3% DMSO solution s.c)

Metabolomics profile confirmed alteration of cellular proteins at the molecular level. Therefore, in the subsequent step, we performed western blotting of major proteins to validate the above findings. MNU administration significantly increased the level of HIF-1α, SREBP-1c, and FASN and decreased the expression of PHD-2 when the trend was compared with normal control, toxic control, and treatment groups. Treatment with low and high VOA doses significantly reduced the expression of HIF-1α, SREBP-1c, and FASN compared with toxic control animals (Fig. 8). An increase in PHD-2 expression was observed only with a low dose, but no significant PHD-2 expression was observed with a high dose. Treatment with TMX also enhanced the PHD-2 expression and reduced the HIF-1α, SREBP-1c, and FASN levels. Combination therapy with VOA and TMX worked well and significantly reduced HIF-1α, SREBP-1c, and FASN and enhanced PHD-2 expression. No change in the above proteins was noted in DMSO-treated animals, indicating that DMSO does not interfere with protein expression at the molecular level.

## Discussion

MNU is a well-known carcinogenic agent for the induction of mammary gland carcinoma. Being an alkylating agent, it binds with the guanine base pair of DNA and forms two intermediate compounds, namely N-methyl guanine and O-methyl guanine. Methylations of the O-position of guanine alter its hydrogen bonding properties and ultimately damage the DNA of mammary glands cells [21]. The cells with damaged DNA start dividing in an uncontrolled way and develop hypoxia as they move away from blood vessels.

Hypoxia activates HIF-1α in solid tumors of mammary glands, which helps in the furbishing of cancer cells even in oxygen scarcity [22]. It guards the glucose metabolism chiefly through glycolysis which benefits the cancer cells in various ways, as discussed above [23, 24]. PHD-2 is a negative regulator of HIF-1α, which becomes inactive in a hypoxic environment, and its chemical activation can retrograde all its effects along with inhibition of fatty acid synthesis [25]. Previously, our research group proved that PHD-2 activation by BBAP-1, BBAP-2, and BBAPH-1 (compounds of synthetic origins) downregulated HIF-1α and reduced expression of FASN in ER+ mammary gland carcinoma [25–27].

In this study, we selected VOA (a compound of natural origin) for the activation of PHD-2 and consequently down-regulation of HIF-1α guided fatty acid synthesis to fight mammary gland carcinoma based on in silico docking studies.

The in silico ADME studies have shown that the drug is poorly water-soluble; therefore, the drug solution was formed in DMSO (3%). As per the in silico findings, the drug VOA is an inhibitor of CYP2C19 and CYP2C9 and substrate of CYP3A4 liver enzymes, indicating that it can cause liver toxicity as well enhance the toxicity of other drugs inhibited by above said enzymes (Table 1). The drug VOA inhibited ether ether-a-go-go related gene (hERGI&II), which belongs to the multigenic family of voltage-activated, outward rectifying K^+^ channels and mediate the physiological potassium currents during the repolarization phase of cardiac action potential [28]. These findings suggest that VOA can cause some degree of cardiotoxicity. The drug also shown positivity for the Ames test, which warns against its mutagenic potential [29].

Further, outcomes of in silico findings were validated with in vivo studies. First of all cardiotoxic potential of VOA and TMX was evaluated. The perturbed ECG changes like PR interval, Q amplitude, HR, ST height, JT interval, R amplitude, QT interval, T amplitude, and QTc interval observed after MNU administration were largely restored normal with VOA low dose (Fig. 2**,** Table 2**).** This indicates VOA at a low dose is safe for cardiac patients, but this is not true with a high dose of VOA.

HIF-1α promotes neovascularization in solid tumors to accomplish nutrient and oxygen supply to the rapidly dividing cancer cells [7, 30]. According to the previous studies, excessive angiogenesis was observed after MNU treatment characterized by ab, lo, teb and lateral buds. Further increase in blood vessel formation was terminative upon treatment with low dose VOA and TMX. It would be pertinent to mention that better anti-angiogenetic action was observed with a low dose of VOA (Fig. 3G1-G7).

Histopathology of mammary gland tissue was further performed to confirm the morphological changes in MNU treated rats. Extreme damage to dld and distortion of ad was observed, corresponding to the previous studies [31]. No lolular and ab formation along with ted, teb like MNU treated rats were seen after treatment with VOA, and TMX therapy, Better protection to mammary gland was impacted by the low dose of VOA and combination therapy (VOA + TMX) as evidenced by the lower number of teb, ted, lb. No lo formation was noted in these animals (Fig. 3G1-G7).

Cachexia in advanced cancers of the mammary gland is another encountered problem, and various studies have reported a progressive weight loss in cancer [32, 33]. Lee et al. said that loss of body weight in cancer is different from those faced by the people on hunger strike [34]. Similar results were also observed in the current study after treatment with MNU. MNU treated rats documented a constant decrease in body weight, consistent with the previous studies (Fig. 4). The low dose of VOA, TMX, and combination therapy prevented further decrease in body weight. Still, excessive body weight loss like toxic control was observed with a high dose of VOA, indicating its toxicity at high dose.

Mammary gland carcinogenesis is associated with free radical oxidative stress. SOD, Catalase, and GSH are family enzymes that work in tandem to fight against this oxidative stress. Excess of H_2_O_2_ produced in breast cancer may be due to the increased production of superoxide anions (O^2−^) [35]. MNU treatment decreased the enzymatic activity of SOD, GSH, and Catalase which indicates the development of oxidative stress, and VOA and TMX restored the enzymatic back to normal (Table 3). Increased activity of TBARs and PC is described as the indicator of oxidation of lipid membrane. The same was also observed in the current study, and the level of same was also restored to normal upon initiation of therapy with VOA and TMX.

**Fig. 9:**
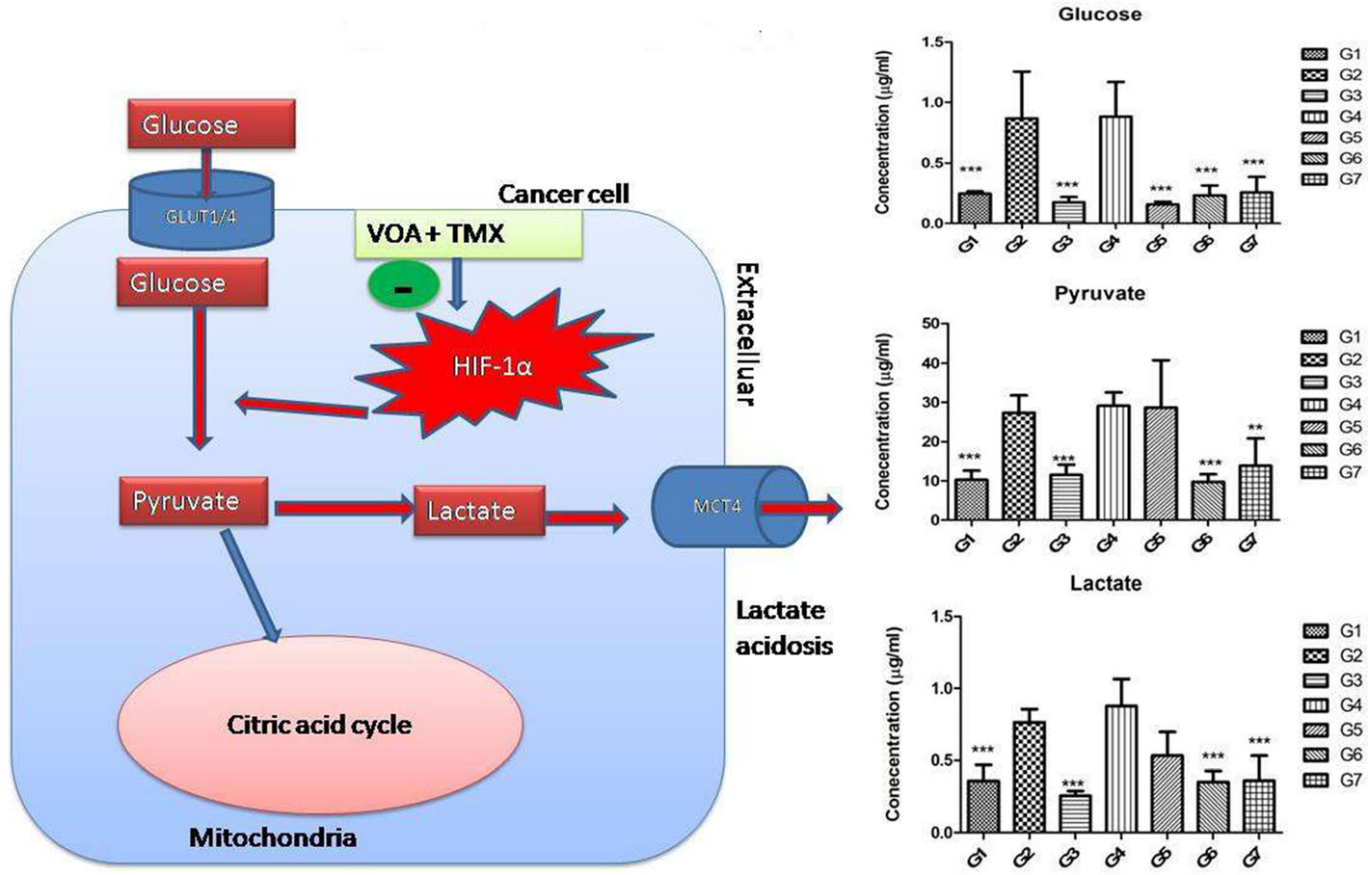
Effect of VOA/TMX therapy on hypoxia-induced metabolic reprogramming of serum metabolites involved in glucose metabolism. In response to hypoxia, cancer cells utilize more and more glucose which is not used in oxidative phosphorylation in mitochondria, and as a result, excess pyruvate accumulates in the cells. Accumulated pyruvate is then converted into lactate which is expelled out through MCT4 transporter into the tumor microenvironment and makes the extracellular environment exploited in various ways by the cancer cell. Treatment with VOA/TMX caused activation of PHD-2, which carries out the hydroxylation and finally proteolytic degradation of HIF-1α. Inactivation of HIF-1α ultimately reduced the glucose utilization and lactate acidosis in the tumor microenvironment. G1(normal control receives normal saline 2 ml/kg, p.o.); G2 (toxic control receives MNU 50 mg/kg, i.p); G3(MNU 50 mg/kg, i.p. + VOA 1 mg/kg, s.c.); G4 (MNU 50 mg/kg, i.p. + VOA 2 mg/kg, s,c.); G5 (MNU 50 mg/kg, i.p. + TMX 8 mg/kg, p.o.); G6 (MNU 50 mg/kg,i.p. + VOA 1 mg/kg, s.c. + TMX 1 mg/kg s.c.) and G7 (dummy control receives 3% DMSO solution s.c)

Perturbation in serum metabolites is the hallmark of cancer [36]. It was previously reported that hypoxia-activated HIF-1α reprograms the metabolism of glucose, fatty acids, and amino acids. A similar type of perturbation was also observed in the current study. An increase in glucose, pyruvate, and lactate in serum of toxic control evidenced that glucose metabolism was only carried out through glycolysis (Fig. 9). Upon treatment with VOA low dose, TMX and both of these drugs decreased glucose, pyruvate, lactate and restored hypoxia-regulated glucose metabolism. Also, high glucose, pyruvate, and lactate were observed in VOA in high dose treated groups that pointed to toxicity VOA.

**Fig. 10:**
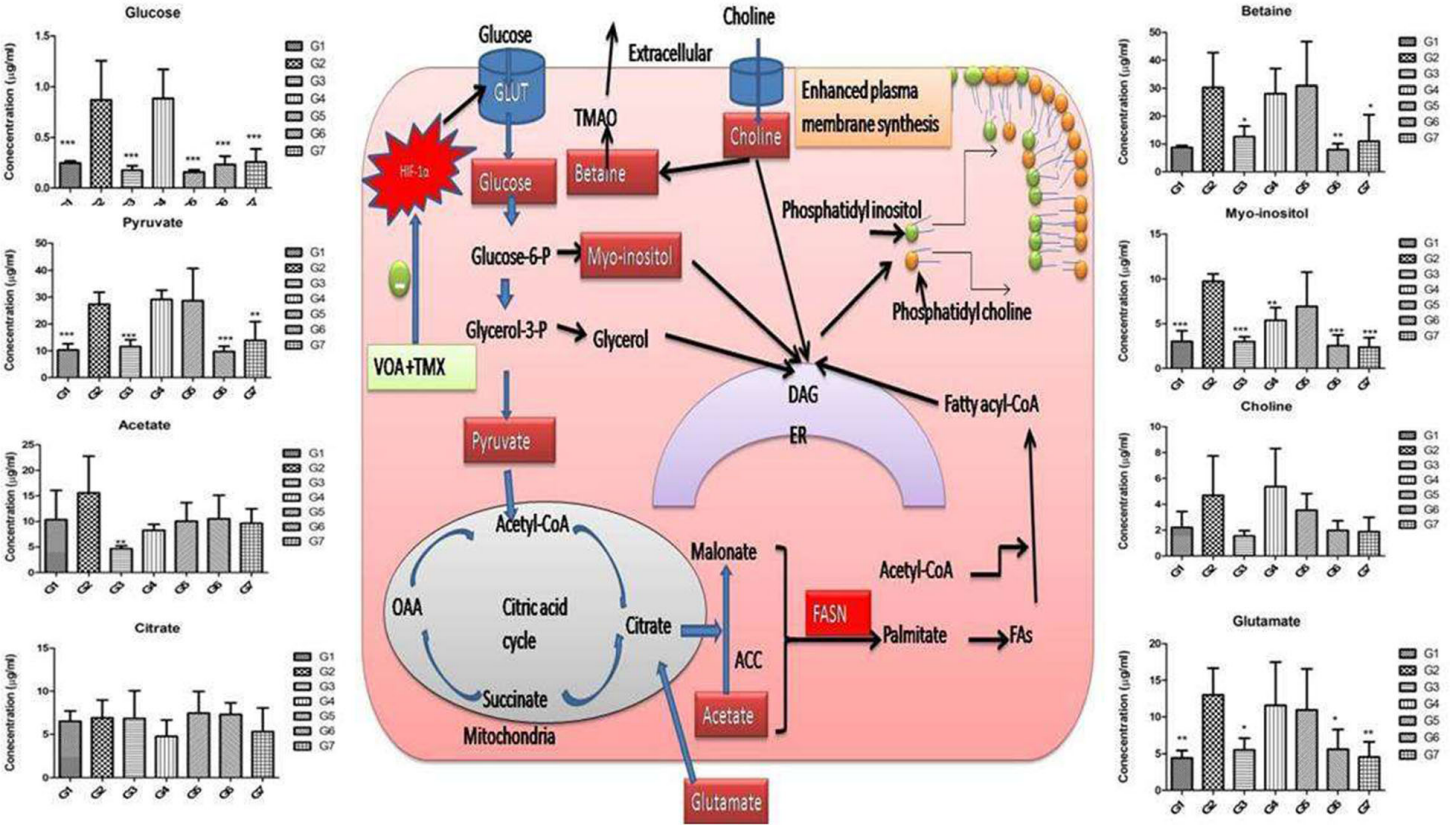
Effect of VOA/TMX on hypoxia-induced metabolic reprogramming of serum fatty acids. Cancer cells remain in high demand of fatty acids required for plasma membrane synthesis of cancer cells. Consequently, glycolysis is upregulated to enhance de novo fatty acid synthesis. Glutamate from extracellular regions also helps in de novo fatty acid synthesis. To form plasma phospholipids, choline, and myo-inositol processes in the endoplasmic reticulum. Phosphatidylcholine and phosphatidylinositol thus formed incorporated into the newly synthesized plasma membrane. Glycerol and myo-inositol are supplied by glycolysis, and choline is supplied from the dietary sources or degradation of plasma lipids. Treatment with low dose of VOA, and its combination with TMX, restored the perturbed metabolic profile of experimental animals. Also, metabolic profiles like that toxic control animals were observed in VOA high dose and with monotherapy of TMX

Various studies have reported that lactate acidosis accomplishes many functions for cancer cells. Its role in altering fatty acid metabolism, induction of angiogenesis, invasiveness is already reported in previous studies [37]. Angiogenesis is necessary for new blood vessel formation to re-establish nutrient supply and enhanced fatty acid for plasma membrane synthesis of rapidly dividing cancer cells [38]. Enhanced angiogenesis in toxic control already discussed in the above section that the lactate acidosis might initiate. Abrupt fatty acid synthesis in response to hypoxia is previously reported in numerous studies [39, 40]. In this study, serum metabolomics analysis of experimental rats has shown an upsurge level of choline, betaine, myo-inositol, acetate, glucose, pyruvate, and lactate, which validates the role of hypoxia in the reprogramming of fatty acids (Fig. 10). To synthesize plasma membrane, phospholipids like-phosphatidylcholine, and phosphatidylinositol, choline and myo-inositol must be present in adequate amounts. Choline is supplied through dietary sources and catabolism of body phospholipids, while glycolysis provides glycerol and myo-inositol; this is why cancer cells guard glucose metabolism, mainly glycolytic pathway. To be converted into phospholipids, fatty acids, glycerol, choline and myo-inositol accumulate on the cytosolic side of the endoplasmic reticulum and newly synthesized phospholipids are incorporated into the plasma membrane of growing cells (Fig. 10). The increased level of above said metabolites in response to hypoxia is line with the previous findings. It would be pertinent to mention here that treatment with VOA low dose and combination with TMX worked well to re-establish the metabolic profile of fatty acid.

**Fig. 11:**
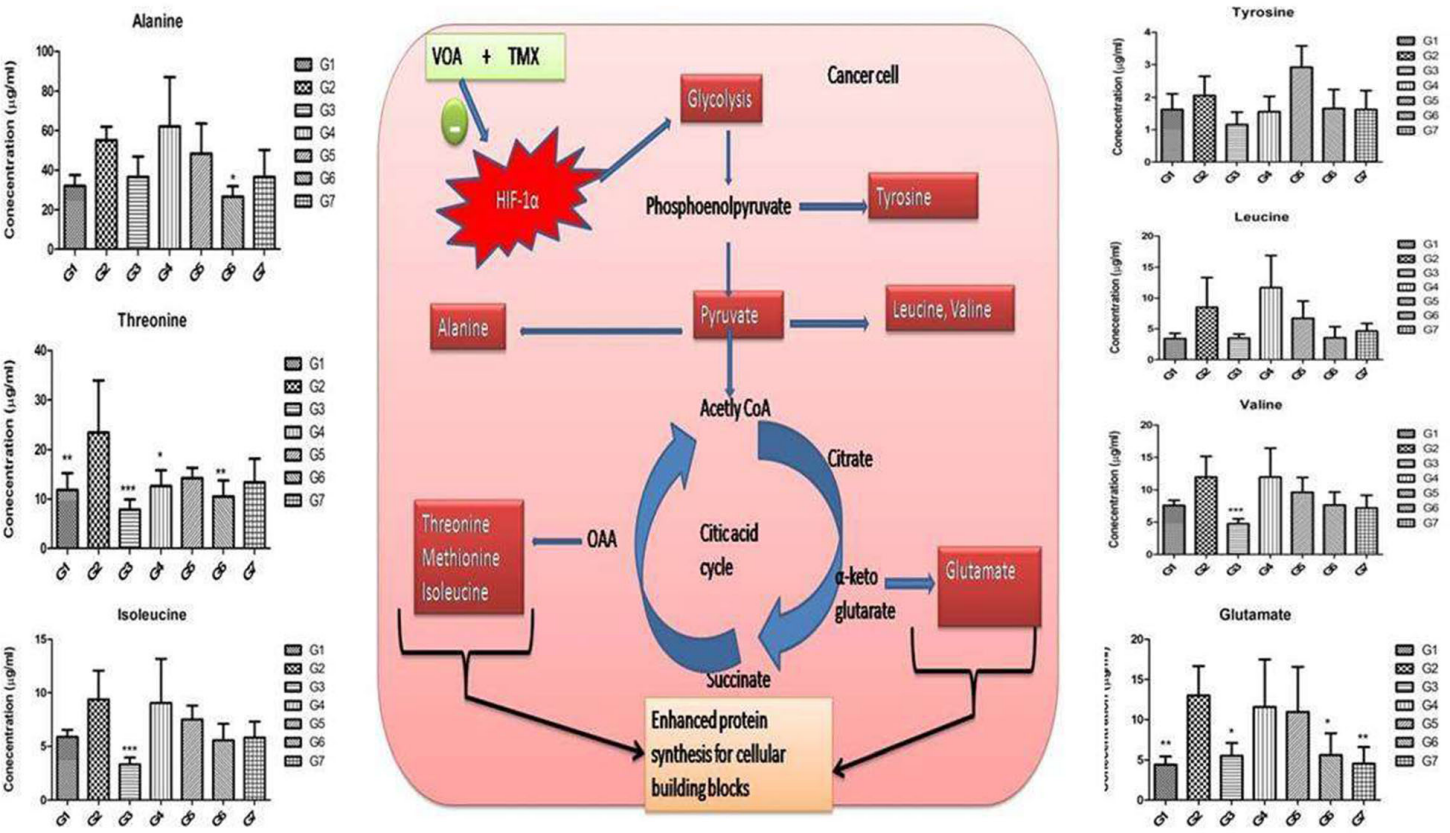
Effect of VOA /TMX therapy on hypoxia-induced metabolic reprogramming of serum amino acids. After MNU administration, hypoxia developed in the cancer cells, which enhanced the glycolytic pathways. As a result of this, amino acid metabolism was reprogrammed. Due to the increase in biosynthesis of amino acids like alanine, threonine, tyrosine, leucine, isoleucine, and glutamate, excess polypeptides are formed to be incorporated into the plasma membrane of rapidly dividing cells. Glutamate also acts as substrates in fatty acid synthesis. G1(normal control receives normal saline 2 ml/kg, p.o.); G2 (toxic control receives MNU 50 mg/kg, i.p); G3(MNU 50 mg/kg, i.p. + VOA 1 mg/kg, s.c.); G4 (MNU 50 mg/kg, i.p. + VOA 2 mg/kg, s,c.); G5 (MNU 50 mg/kg, i.p. + TMX 8 mg/kg, p.o.); G6 (MNU 50 mg/kg,i.p. + VOA 1 mg/kg, s.c. + TMX 1 mg/kg s.c.) and G7 (dummy control receives 3% DMSO solution s.c)

Along with fatty acids, the demand for amino acids is increases parallely and equally important in cancer cells needed for the plasma membrane proteins, enzymes, and signaling molecules. Reprogramming in serum amino acids like threonine, isoleucine, leucine, tyrosine, glutamine, and alanine was observed in response to hypoxia in the current study after MNU administration (Fig. 11). Low dose VOA and its combination therapy restored the perturbed amino acid metabolism. The pooled analysis of serum metabolites involved in the glycolytic pathway, protein synthesis pathways, and fatty acid synthesis pathway shows that cancer cell speeds up glucose metabolism to meet increasing demands of fatty acids and amino acids needed for plasma membrane synthesis. Treatment with a low dose of VOA and TMX restored the metabolic perturbation to normal. Also, toxic effects rather than favorable outcomes were observed with a high dose of VOA.

Western blotting was performed to evaluate further the effect of the drug at the molecular level. Immunoblotting of previous studies has reported a large increase in cellular expression of HIF-1α, SREBP-1c, FASN, and diminished expression of PHD-2 in solid tumors of the mammary gland [41, 42]. Similar kinds of manifestations were also noted in toxic control after MNU administration, i.e., the increase in HIF-1α, SREBP-1c, FASN but a decrease in PHD-2, which indicated the development of cancer hypoxia in these animals (Fig. 8). The changes described above might be required to meet the high demand of fatty acids to be used for plasma membrane synthesis. On the other hand, the opposite trend in the expression of HIF-1α, SREBP-1c, FASN, and PHD2 indicated that VOA and TMX treatment activated PHD-2, which enhanced the proteolytic degradation of HIF-1α and reverted its downward effects.

## Conclusion

It was concluded from the results of the current study that VOA at a low dose and its combination therapy reduced fatty acid synthesis in MNU induced mammary gland carcinoma of albino Wistar rats. However, a higher dose of VOA caused significant toxicity in the experimental animals.

## Material and methods

### In silico study

#### Scrutiny of compounds

PyRx (https://pyrx.sourceforge.io/) software was used for the virtual screening of compounds. It is free in silico screening software, which is employed for searching the library of drug molecules from databases. Autodock 4.2 was used to determine the strength of interaction between various ligands and targets in combination with 3D visualization. Ligands structure was searched from the ZINC database, and protein structure was searched from Protein Data Bank (PDB id-2G19) [43, 44]. A library of 5000 natural compounds from natural origin was downloaded based on their 60% structure similarity with Vincristine from the ZINC database. All the compounds were filtered by the Lipinski drug-like filter (http://www.niper.gov.in/pi_dev_tools/DruLiToWeb/DruLiTo_index.html), and then individual compound was docked with PHD2 protein to determine the binding energy. Compounds having good binding energy with PHD2 were selected and searched for their availability. Finally, VOA (ZINC169368472) was obtained as a drug sample from National Cancer Institute, USA (https://dtp.cancer.gov/RequestCompounds/index.xhtml). Further, in silico rodent oral toxicity and ADME of VOA were predicted using pkCSM software (http://biosig.unimelb.edu.au/pkcsm/prediction_single/adme_1604411699.32) **(**Table 1**)** [45].

### In vivo study

#### Chemicals and reagents

VOA (NCI-3375-85-5) was obtained from National Cancer Institute (NCI), United States as a drug sample. TMX (Tamodex20-Zydus) was purchased from the local market. DMSO (Merk), ponceauS (Himedia.ML045), hematoxyline (Himedia, So58),), Sodium Lauryl Sulfate (SLS) (Loba chemieS56971301), MNU (Sigma Aldrich,57–97-6), TEMED (Amresco-0761), acrylamide (Genetix-1443c196), Protein assay kit (Amresco, M173), bovine serum albumin (BSA) (Genetix, PG-2330), eosin (Himedia, S007), ammonium persulfate (APS) (Lobachemie-LB2282a09), Glycine (Amresco-0167-kg), TrisUlrapure (DuchefaBiochemie), RIPA lysis buffer (Amresco, N653), transfer buffer (Genetix, GX-9411AR), All other chemicals used in this study were purchased from Genetix Asia Pvt. Ltd. New Delhi, India and all the chemicals were molecular grade.

#### Experimental animals

Female albino Wistar rats in the weight range 60-100 g were procured from Central Drug Research Institute (CDRI) Lucknow (protocol approval No. UIP/IAEC/May-2016/06). After procurement, animals were kept in the quarantine room of the departmental animal house to acclimatize to the laboratory conditions. Tetracycline (500 mg/kg, p.o) was administered to the animals for the next 7 days through water bottles to prevent bacterial infection. Also, 12 h light /dark cycle, constant temperature, and humidity were maintained along with free access to food and water ad libitum. The guidelines laid by the Committee for the Purpose of Control and Supervision of Experiments on Animals (CPCSEA) were followed in the conduction of all experiments. After 1 week, animals were randomly selected (in the weight range 100-150 g) and divided into seven groups (eight animals in each group). G1(normal control receives normal saline 2 ml/kg, p.o.); G2 (toxic control receives MNU 50 mg/kg, i.p); G3(MNU 50 mg/kg, i.p. + VOA 1 mg/kg, s.c.); G4 (MNU 50 mg/kg, i.p. + VOA 2 mg/kg, s,c.); G5 (MNU 50 mg/kg, i.p. + TMX 8 mg/kg, p.o.); G6 (MNU 50 mg/kg,i.p. + VOA 1 mg/kg, s.c. + TMX 1 mg/kg s.c.) and G7 (dummy control receives 3% DMSO solution s.c.). At the time of MNU administration, the animal’s age was 110 days old. In group 6, lower VOA and TMX were selected because both drugs are P-glycoprotein inhibitors (efflux pump), enhancing each other’s intracellular concentration in cancer cells. MNU solution was prepared in 3% DMSO solution and given once at the inception of the experiment. Treatment with VOA/TMX was started 15 days after MNU administration and stopped after 1 month. The experiment was terminated after 100 days. One day before the dissection, ECG changes of the experimental animals were recorded. Next day, animals were anesthetized with cocktail of diazepam (5 mg/kg/i.m.) and ketamine hydrochloride (100 mg/kg/i.m.) [46]. The blood was withdrawn from the retro-orbital plexus to analyze the serum metabolic profile of experimental animals. Afterward, the animals were sacrificed by cervical dislocation, and the abdominal cavity was opened to excise the mammary glands [47]. Excised mammary gland tissue was preserved in the − 20 °C until further analysis.

#### Hemodynamic analysis

Hemodynamic analysis of experimental animals was performed using AD Instrument to assess cardiac toxicity due to MNU and VOA. Purposely, ketamine (50 mg/kg, i.m.) and diazepam (2.5 mg/kg, i.m.) was used to anesthetize the animals and mounted on the wax trays. Platinum hook electrodes were placed at the sterilized dorsal and ventral thorax skin. These electrodes had connections with Bio-amplifier (ML-136) and channel power lab (ML-826, Australia), converting analog signals into digital signals. The ECG signals thus recorded and saved on the hard disk which were analyzed offline using Lab Chart Pro-8 (AD-Instruments, Australia).

In addition to this, the above ECG signals were further used for HRV analysis. Before this, a raw signal was manually inspected for the correct detection of R waves, and from this, HR was calculated by plotting the R waves per unit time. Finally, HRV was calculated using Lab Chart Pro-8 (AD-Instruments) after setting time and frequency parameters [48, 49].

#### Carmine staining

Carmine staining is an excellent method to view angiogenesis in malignant cancers. Mammary gland tissue was carefully removed and stretched over the glass slides, and dried for some time. Afterward, slides were kept in Carnoy’s fixative solution, which was formed by mixing ethanol (60%), chloroform (30%), and glacial acetic acid (10%) continuously for 2 h. Subsequently, slides were washed with decreasing ethanol concentrations (90, 70, 35, and 15%) for 1 h and then placed in carmine staining solution for 2 days. Carmine staining solution was formed by dissolving 1 g carmine and 2.5 g aluminum potassium sulfate in 500 ml water. After 2 days, slides were dehydrated again with dipped in increasing ethanol concentration (70, 95, and 100%) and then placed in the xylene to remove the lipid adhere on the tissue for an overnight period. Finally, prepared slides were examined under the biological microscope, and images were taken at 4X resolution [50, 51].

#### Histopathological analysis

To examine the morphology of the mammary gland, small tissue sections were locked in formaldehyde (10%) solution and then planted in the wax cubes. Afterward, 5 μm sections were sliced with the microtome and stained with hematoxylin and eosin (H&E). Finally, stained tissue sections were visualized and photographed at 4X using a digital biological microscope (BR-Biochem Life Sciences, N120, New Delhi, India) [52, 53].

#### Weight variation

A constant decrease in body weight has been reported in cancer patients [54]. In the current study, initial body weight and final body of control, toxic and treated groups were measured. Percentage body weight was calculated the below-given formulae:-.

Percentage (%) decrease in body weight = (Final body weight-initial body weight/Initial body weight) X100.

#### Antioxidant markers

Tissue samples from each group were accurately quantified, and tissue homogenization was prepared in KCl (0.15). Subsequently, the tissue samples were centrifuged for 15 min at 10000 g to get supernatants. The supernatants thus obtained stored in ice-cold water until further analysis. At the time of analysis, a fraction of the supernatant was taken and used to determine SOD, catalase, TBARs, PC, and GSH according to the previous method described elsewhere. The absorbance of the samples was taken through a UV-Visible spectrophotometer (Agilent Technologies, Carry 60) [55, 56].

#### ^1^H NMR study

The serum metabolic profile of experimental rats was analyzed with a Bruker NMR spectrometer (Avance-III) operating at a frequency of 800 MHz equipped with Cryoprobe. Serum samples were prepared by taking 220 μl of serum in NMR tubes (5 mm) (Willmadglasss, USA), and the final volume is adjusted to 440 μl with the addition of 220 μl of NMR buffer solution, which was prepared in D_2_O by adding sodium phosphate saline of strength 20 mm (pH 7.4). Into this, another tube termed as co-axial with diameter 2 mm containing 0.5 mM solution of 3-trimethylsilyl-(2,2,3,3-d4)-propionic acid (TSP) was inserted. This acted as an external lock and standard for the NMR experiments concerning metabolic quantification and assignment. After that, resultant NMR tubes were placed in the analysis point, and raw NMR spectra were recorded and stored on the hard disk. The RAW NMR data thus obtained and analyzed with Bruker software Topspin-v2.1 (Bruker BioSpinGmb H, Silberstreinfen 476,287 Rheinstetten Germany). The spectra corresponding to the signals from lipoproteins, glycoproteins, amino acid, lactate, and choline were mainly focused. The CPMG (0.7–8.6) spectra were binned and integrated using the AMIX package (Version3.9.15, Bruker). Further, the concentration of individual metabolites was determined with Chenomax software [42, 47].

#### Western blotting

A tissue sample (500 g) was prepared in RIPA lysis buffer and phenylmethylsulfonyl fluoride (PMSF). After protein quantification with Bradford assay, 12.5% SDS-PAGE gel was employed to separate the proteins according to the principle of Laemmle. The separated proteins were transferred onto the PVDF membrane (IPVH00010 Millipore, Bedford, MA USA). Blocking of the transferred proteins was done with a mixture of 5% BSA and 5% nonfat skimmed milk prepared in TBST. After 3 h blocking, the membrane was incubated with primary antibodies like FASN (SC-55580), SREBP-1c (SC-13551), PHD-2 (SC-67030), HIF-1α (SC-13515) overnight and then incubated with HRP conjugated secondary antibody [anti-goat (SC-2020)]at room temperature for 3 h [57]. In the last, blots were developed with chemiluminescence substrate (Western Bright ECL HRP substrate Advanta, Melanopark, California, US) in ChemiDoc XRS+ (Bio-Rad) after washing one time with TBST. β-actin was used as a reference standard in the analysis of protein blots.

### Statistical analysis

The whole data is presented as mean ± SD. To identify the possible significance, Mean and SD were further analyzed by one-way ANOVA (followed by Bonferroni test). The values *p < 0.05, **p < 0.01, ***p < 0.001 were considered as statistically significant. Statistical analysis was performed using Graph Pad Prism software (5.02).

## Data Availability

The datasets used and analyzed during the current study are available from the corresponding author on reasonable request.

## Abbreviations

TMX: Tamoxifen
ER: Estrogen receptors
FASN: Fatty acid synthase
HIF-1α: Hypoxia-inducible factor-1α
PHD-2: Prolyl hydroxylase-2
VOA: Voacamine
ECG: Electrocardiogram
DMSO: Dimethyl sulphoxide
HRV: Heart rate variability
NMR: Nuclear magnetic resonance
lo: Lobules
ab: Alveolar buds
ted: Terminal end duct
lb.: Lateral bud
tb: Terminal bud
MNU: N-methyl-n-nitrosourea
TBARs: Thiobarbituric acid reactive substances
PC: Protein carbonyl
SOD: Superoxide dismutase
GSH: Glutathione
APS: Ammonium persulfate
SLS: Sodium Lauryl Sulfate
BSA: Bovine serum albumin
PMSF: Phenylmethylsulfonyl fluoride
H&E: Hematoxyline and eosine
